# Disentangling binge eating disorder and food addiction: a systematic review and meta-analysis

**DOI:** 10.1007/s40519-021-01354-7

**Published:** 2022-01-18

**Authors:** Ester di Giacomo, Francesca Aliberti, Francesca Pescatore, Mario Santorelli, Rodolfo Pessina, Valeria Placenti, Fabrizia Colmegna, Massimo Clerici

**Affiliations:** 1 School of Medicine and Surgery, University of Milano Bicocca, Milano, Italy; 2 Psychiatric Department-ASST Monza, Monza, Italy

**Keywords:** Food addiction, Binge eating, Obesity, Eating disorders, Bulimia nervosa

## Abstract

**Background and aims:**

The concept of "Food Addiction" has been based on criteria of Substance Use Disorder. Several studies suggested a relationship between food addiction and eating disorders, but little is known about its extent or role.

We aim at exploring if food addiction is coincident with a specific eating disorder (binge eating disorder appears the closest) or it is a separate diagnostic entity that afflicts in comorbidity with eating disorders or other conditions like obesity or even in the general population.

**Methods:**

This systematic review and meta-analysis analyzed observational studies with a comparative estimation on rates of subjects affected by binge eating disorder and food addiction.

**Results:**

Binge eating disorder shows higher comorbidity with food addiction compared to other eating disorders (OR = 1.33, 95% CI, 0.64–2.76; c^2^ = 4.42; *p* = 0.44;I^2^ = 0%), or each eating disorder [anorexia nervosa purging type (OR = 1.93, 95% CI, 0.20–18.92; *p* = 0.57) and restrictive type (OR = 8.75, 95% CI, 1.08–70.70; *p* = 0.04)], obese patients (OR = 5.72, 95% CI, 3.25–10.09; *p* =  < 0.0001) and individuals from the general population (OR = 55.41, 95% CI, 8.16–376.10; c^2^ = 18.50; *p* < 0.0001; *I*^2^ = 0%)but has decreased prevalence when compared to bulimia nervosa (OR = 0.85, 95% CI, 0.33–2.22; c^2^ = 0.35; *p* = 0.74; *I*^2^ = 0%).

**Discussion and conclusions:**

Our data show that the prevalence of food addiction in binge eating disorder is higher than in other eating disorders except in bulimia nervosa. Moreover, it is a separate diagnostic reality and can be detected in people without mental illness and in the general population.

Food addiction might have a prognostic value, since in comorbidity, and should be addressed to boost treatment efficacy and patient’s recovery.

**Level of evidence:**

I: Evidence obtained systematic reviews and meta-analyses.

## Background

Overeating, obesity and eating disorders are serious issues of the modern wealthy societies worldwide.

The reasons why some people chronically overeat and are unable to limit their food intake have been examined. A growing number of clinical and neurobiological evidence has shown that, in vulnerable subjects, persistent overeating may lead to a pattern of compulsive behavior similar to that of drug abuse and other addictive disorders  [1–4].

The need of a better definition of this behavioral model led to the formulation of the concept of "Food Addiction" which gained a growing interest in the scientific literature focused on eating disorders with particular attention to the underlying neurobiological and phenomenological mechanisms [5].

The term "Food Addiction" has been built on behavior and subjective experiences related to food consumption that resemble criteria of Substance Use Disorder (SUD) (e.g., strong urge to consume, exacerbated by abstinence, and failure to limit consumption despite the awareness of toxicity)  [6].

From a biological perspective, many neurobiochemical and neurogenetic studies proved typical mechanisms of addictions are crucial in problematic nutrition. Genetic similarities between overeating and substance addiction are variants in the genes encoding the dopamine D2 receptor allele of the ANKKI gene (e.g., Taq1 (rs1800497) and transporter (e.g., SLC6A3, DAT1) and opioid receptor genes (OPRM1 gene) [7]. Furthermore, illicit drugs and food exert the same gratifying effect through the activation of dopamine neurons in the ventral tegmental area with consequent release of dopamine in the nucleus accumbens and effects on the mesolimbic pathway  [8].

Although the definition of Food Addiction (FA) is not yet categorized in the Diagnostic and Statistical Manual of Mental Disorders, the academic debate on a possible addictive potential of food keeps going [9]. In fact, many overlapping criteria with the DSM-5 drug addiction include: (1) substance taken in larger quantities and for a longer period than expected; (2) persistent desire or repeated failed attempts to quit; (3) a large amount of time and energy spent on obtaining, using or disposing of substances; (4) reduction of important social, occupational, or recreational activities because of substance use; (5) continued use despite knowledge of the negative consequences; (6) tolerance; and (7) withdrawal symptoms  [10].

## The Yale Food Addiction Scale (YFAS)

The Yale Food Addiction Scale (YFAS) has been developed by Gearhardt [10], as a first categorical attempt of the concept of FA.

The YFAS is a 25-item self-report questionnaire that adapts the diagnostic criteria of substance dependence of the DSM IV-TR to eating behavior or abuse of specific foods. Currently, it is the only validated tool for assessing symptoms of "food addiction".

It has several categories of scoring: symptoms count (seven diagnostic criteria) and a diagnostic threshold that reflects the criteria for the diagnosis of substance dependence (the presence of three or more symptoms plus clinically significant impairment or distress).

Up to 11.4% of the general population may show symptoms of food addiction, while approximately 25–42% of obese patients meet the YFAS criteria [11].

### The concept of binge eating

Binge eating disorder (BED) is an eating disorder characterized by recurrent episodes of bingeing, in the absence of compensatory behaviors (purging, overexercize etc.). [12].

A binge episode is defined as eating an unusually large amount of food over a discrete period of time associated with a subjective sense of loss of control while eating  [13].

The presence of BED in individuals with obesity has been associated with a significant increase in the risk of psychosocial, psychiatric, and medical problems [14].

It is estimated that 15.7% to 40% of obese patients suffer from BED [15, 16] as well as between 1.12% and 6.6% of the general population  [17, 18].

The Binge Eating Scale (BES) is the basic tool used to confirm a clinical diagnosis of BED [18].

### Association/overlaps between BED and FA

As mentioned above, phenotypic overlaps have been observed between the definition of food addiction and binge eating disorder [19].

Both BED and food addiction are characterized by the loss of control over consumption, continued overuse despite negative consequences, and repeated failed attempts to reduce consumption. Because of these similarities, measures of binge eating disorder (BED) and food addiction (YFAS) are often highly correlated, highlighting difficulties in assessing and distinguishing those two different constructs [20, 21]. Some preliminary evidence reporting high frequencies of FA in patients with BED suggests that the co-occurrence of BED and FA may represent a more disturbed BED subgroup  [22].

Emerging research suggests that binge eating and food addiction, while sharing many characteristics, may have important distinctions. In samples of individuals with BED, the frequency of food addiction ranges from 42 to 57%, suggesting that, despite the multiple similarities, the constructs do not completely overlap. Furthermore, in the study by Gerhardt et al., authors show that, compared to patients with BED without food addiction, participants with BED and food addiction had significantly higher levels of low self-esteem, depression, negative affect, emotional dysregulation, but they did not show significantly different levels of dietary moderation. A link between the severity of food addiction and eating disorders was, therefore, hypothesized, providing FA with a prognostic value  [10, 20].

### Food addiction and other eating disorders (EDs)

Food addiction shows significant diagnostic overlaps with other EDs as well, especially with Bulimia Nervosa (BN), an eating disorder characterized by the presence of binge eating episodes with compensatory behaviors that do not occur in BED. Furthermore, BN is linked to impaired reward sensitivity in dopaminergic brain circuit, increasing the addictive potential [23]. Moreover, some studies emphasize that many patients affected by BN may have a body weight within the normal range but may also be addicted to food [24, 25]. Additionally, even if without a systematic evaluation, some studies attest an association between FA and BN ranging from 81 to 97% [26, 27]. Even with fundamental differences, in fact, common overlapping symptoms between FA, BED and BN exist (e.g., reduced feeding control, continued use despite negative consequences)  [1, 11].

An interesting further point ascribes food restriction, typical of anorexia nervosa (AN) to addictive behaviors (or "hunger addiction") [28]. Szmukler e Tamtam suggest that "patients with AN are dependent on the psychological and possibly physiological effects of starvation. Increased weight loss results from tolerance to starvation necessitating greater restriction of food to obtain the desired effect, and the later development of unpleasant ‘withdrawal’ symptoms on eating” [29].

The existence of food addiction as a separate entity, as well as its role in several eating disorders is still unclear and poor explored. The aim of the present systematic review and meta-analysis is to attest to the prevalence of FA in different eating disorders and its existence.

## Methods

This systematic review and meta-analysis was performed according to the Meta-analysis of Observational Studies in Epidemiology guidelines [30]. Procedures and study inclusion criteria were defined a priori and registered in PROSPERO (an international prospective register of systematic reviews-CDR42020215998).

### Data sources and search strategy

A systematic search for articles published in electronic databases (PubMed, Embase and PsychINFO) through September 24, 2020, was performed with no time or language restrictions. The selected databases report documents whose titles and abstracts are in written in fluent English, sometimes together with titles and abstracts in authors’ native language. The manuscript main text might be in English or in authors’ native language, depending from each journal rules. Our search produced five results whose main text was not written in English but their titles/abstracts entailed their exclusion according to inclusion/exclusion criteria. Search phrases combined thesaurus and free-search indexing terms related to eating disorder and addiction, using combinations of the following search terms: *binge eating disorder, food addiction*. We contacted corresponding authors of selected studies if additional information was required.

### Eligibility criteria

We included observational studies with a comparative estimation on rates of subjects affected by binge eating disorder and food addiction. We included studies that defined binge eating disorder according to the *Diagnostic and Statistical Manual of Mental Disorders, Fifth Edition* criteria. We excluded studies without an appropriate comparison group, those that did not clearly define food addiction, and case reports. To reduce the risk of misclassification errors, we included only high-quality studies, with unequivocal definitions. If data from the same sample were published in multiple works, we retained only the studies published in peer-reviewed journals, excluding conference abstracts and dissertations.

### Terms and definitions

According to Randolph, who introduced the concept in 1956, the term “Food Addiction” refers to specific food related behaviors characterized by excessive and dysregulated consumption of palatable and high calorie food.

More recently, food addiction has been defined as a chronic, relapsing condition caused by the interaction of many complex variables that increase the desire for certain specific foods to achieve a state of elevated pleasure, energy or arousal, or to alleviate negative emotional or physical states, coming closer to the criteria defining addictive disorders [11, 31].

A food addiction or eating addiction is characterized by the compulsive consumption of palatable (e.g., high fat and high sugar) foods which markedly activate the reward system in humans and other animals despite adverse consequences.

### Data collection process

Three authors (E.d.G, F.P., and F.A.) preliminarily reviewed titles and abstracts of traced articles. The initial screening was followed by the analysis of full texts to check compatibility regarding inclusion and exclusion criteria. Discordances were analyzed and disagreements were resolved by discussion among all the authors. When reported information was unclear or ambiguous or numerical data were not obtainable by percentages, the relevant corresponding author was contacted for clarification.

### Data extraction

A standardized form was used to extract data, including information on year of publication, country, setting, characteristics of study participants (sample size, age, and percentages of men and women), eating disorders, and food addiction. If raw numerical data were not reported, they were calculated by percentages, deriving crude odds ratios (ORs). Two authors (E.d.G. and F.A.) conducted data extraction independently; extraction sheets for each study were cross-checked for consistency, and any differences were resolved by discussion among the coauthors.

### Statistical analysis

Meta-analysis of the overall comparison of food addiction rates among subjects affected by eating disorders and each type of eating disorder compared with binge eating was performed, and pooled ORs with 95% CIs were generated using inversed variance models (DerSimonian–Laird) with random effects [32, 33]. Results were summarized using conventional forest plots. Standard χ2 tests and the *I*^2^ statistic (i.e., the percentage of variability in prevalence estimates attributable to heterogeneity rather than sampling error or chance, with values ≥ 75% indicating high heterogeneity) were used to assess between-study heterogeneity [34]. To test for publication bias, we performed funnel plot analysis and the Egger test on all studies stratified by eating disorder (bulimia nervosa, other eating disorders) and general population. Thus, three separate Egger tests were performed. The Egger test quantifies bias captured in the funnel plot analysis with linear regression using the value of effect sizes and their precision (SE) and assumes that the quality of study conduct is independent of study size. If analyses showed a significant risk of publication bias, we would use the trim and fill method to estimate the number of missing studies and the adjusted effect size [35–37]. Meta-regression analysis was performed to examine sources of between-study heterogeneity if of a high level (*I*^2^  > 75%) on a range of study prespecified characteristics (i.e., sample size, age, and country).

All analyses were performed using R, version 3.2.3 (meta and metaphor packages; R Foundation for Statistical Computing). Statistical tests were two sided and used a significance threshold of *P* < 0.05.

## Results

### Study characteristics

**Fig. 1:**
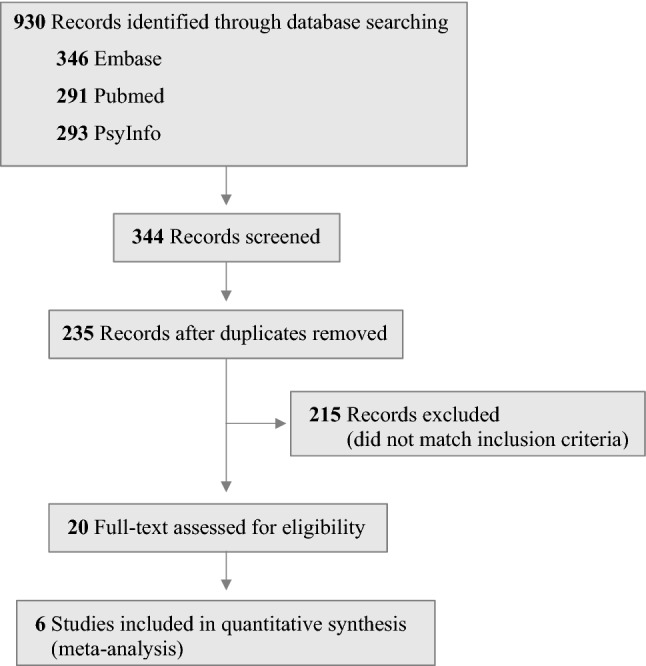
Preferred reporting items for meta-analyses flow diagram

Six studies that involved a total of 2476 subjects (539 affected by binge eating disorder, 178 by bulimia nervosa, 18 by eating disorder NOS, 65 by anorexia nervosa restrictive, 33 by anorexia nervosa purging, 442 subjects with obesity and 1146 people from the general population without eating disorders) were included in the analysis (see Fig. 1). The studies were conducted in 5 countries (Australia, Canada, France, Spain and the United States). All articles were published after 2014 (one in 2014, two in 2017, two in 2019 and one in 2020). Most of the studies had sample weight of less than 10%, whereas one study had sample weight of 20% and one higher than 50%. All study characteristics are summarized in Table 1.

**Table 1:** Characteristics of the studies included in the meta-analysis

Source, Year (Country)	Sample Size Weight	EDs (N)					No EDs (N)	
		BED	BN	ED_NOS	AN-R	AN-P	Obese	Gen_Pop
Burrows 2017 (Australia)	0.543	351						994
Carter 2019 (Canada)	0.057	71						70
Fauconnier 2020 (France)	0.078	15	82		65	33		
Ivezaj et al. 2017 (USA)	0.203	60					442	
Romero et al. 2019 (Spain)	0.029	29	42					
Roser Granero 2014 (Spain)	0.067	13	54	18				82

*EDs* eating disorders, *BED* binge eating disorder, *BN* bulimia nervosa, (*ED_NOS*) eating disorder not otherwise specified, (*AN-R*) anorexia nervosa restrictive, (*AN-P*) anorexia nervosa purging, *Gen_Pop* general population

### Prevalence of food addiction in binge eating disorder compared to other eating disorders

**Fig. 2:**
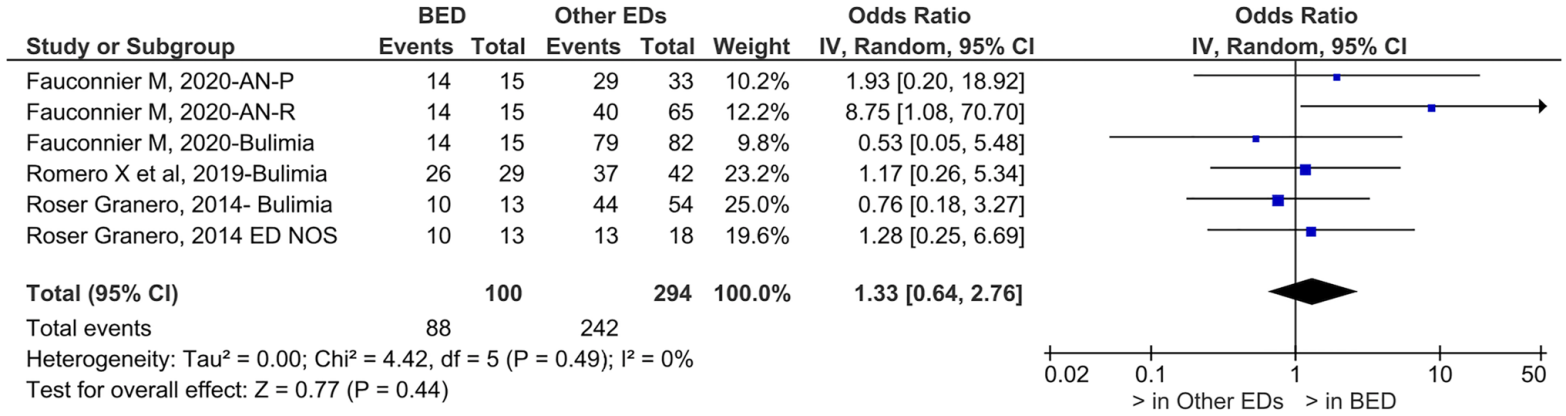
Prevalence of food addiction in binge eating disorder and other eating disorders. *FA* food addiction, *BED* binge eating disorder, *EDs* other eating disorders

Patients affected by binge eating disorder have an increased prevalence of food addiction compared to patients with other eating disorders (OR = 1.33, 95% CI, 0.64–2.76; c^2^ = 4.42; *p* = 0.44; *I*^2^ = 0%)(see Fig. 2).

### Prevalence of food addiction in binge eating disorder compared to each other eating disorder

**Fig. 3:**
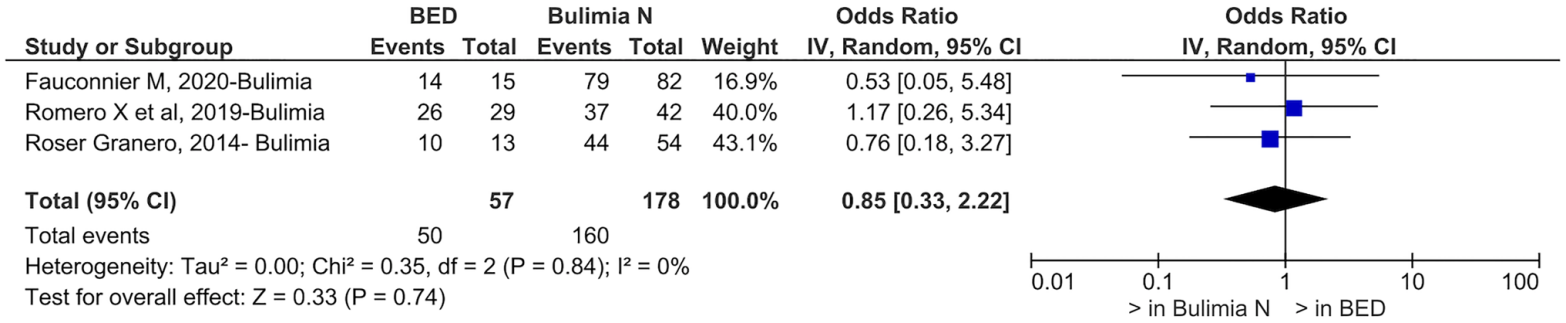
Prevalence of food addiction in binge eating disorder and bulimia nervosa. *BED* binge eating disorder, *Bulimia N* Bulimia nervosa

The prevalence of food addiction in patients affected by binge eating disorder is reduced compared to that of patients with bulimia nervosa (OR = 0.85, 95% CI, 0.33–2.22; c^2^ = 0.35; *p* = 0.74; *I*^2^ = 0%)(see Fig. 3) while it is increased compared to anorexia nervosa purging type (OR = 1.93, 95% CI, 0.20–18.92; *p* = 0.57) and restrictive type (OR = 8.75, 95% CI, 1.08–70.70; *p* = 0.04).

### Prevalence of food addiction in binge eating disorder compared to obesity

Patients affected by binge eating disorder have an increased prevalence of food addiction compared to obese patients without any eating disorder (OR = 5.72, 95% CI, 3.25–10.09; *p* =  < 0.0001).

### Prevalence of food addiction in binge eating disorder compared to the general population

**Fig. 4:**
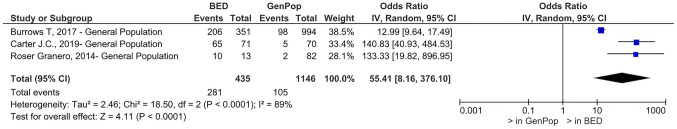
Prevalence of food addiction in binge eating disorder and the general population. *BED* binge eating disorder, *GenPop* general population

Patients affected by binge eating disorder have an increased prevalence of food addiction compared to subjects from the general population without eating disorders (OR = 55.41, 95% CI, 8.16–376.10; c^2^ = 18.50; *p* =  < 0.0001; *I*^2^ = 89%)(see Fig. 4).

### Publication bias

There was no evidence of publication bias in studies focused on the bulimia nervosa group (Egger test; SE,  – 1.20; 95% CI,  – 18.01 to 15.60; *P* = 0.26), for studies focused on other EDs (Egger test; SE, 1.65; 95% CI,  – 4.29 to 7.60; *P* = 0.24) and studies focused on the general population (Egger test; SE, 3.77; 95% CI,  – 10.23 to 17.78; *P* = 0.09).

### Sources of heterogeneity

Univariable and multivariable meta-regressions weighted for the study weight were performed on the following variables that were potentially associated with heterogeneity: (1) the country where the study was conducted (2) the year of publication. The mean age of the samples and the percentage of females within each group were not available or calculable in most of the studies.

None of the parameters, including the sample weight, or a combination of parameters was sufficient to explain heterogeneity detected in the studies focused on the general population.

## Discussion

The concept of “Food Addiction” arose thanks to the identification of common features between drug addiction and some behavioral disturbance in food intake. Even if it is not classified in the DSM-5, a diagnostic tool has been developed applying diagnostic criteria of drug addiction to food.

Starting from evidence of a comorbidity between food addiction and eating disorders, this meta-analysis focus on measuring the extent of that comorbidity in each eating disorder. Binge eating disorder was theoretically the closest construct to that of food addiction. On the contrary, data proves that the prevalence of food addiction in binge eating disorder is higher than in other eating disorders except in bulimia nervosa. People affected by bulimia nervosa, in fact, dwell with food addiction more than people with binge eating disorder, even if the result is not statistically significant. Such evidence is fascinating and deserves considerations. First of all, BED, BN and FA have overlapping symptoms that may contribute to those results. More interestingly, some authors supported the description of BN as an eating behavior similar to addiction. [25–27], thus reinforcing the addictive nature of that disorder.

Beyond overlaps that may justify the presence of FA in patients affected by a specific eating disorder or another, it is crucial to underline that FA is detected in subjects without any eating disorder, even if with obesity, but also in subjects from the general population and without any psychiatric issue. Even though the prevalence of FA in those people is sensibly lower, it is observed, encouraging the idea that FA might be a different and separate entity from a specific eating disorder. The presence of an addictive component in some subjects affected by eating disorders might influence their treatments and outcomes if not rightly addressed. A further consideration, which is supportive to the need of recognizing the presence of addictive characteristics in some subjects with eating disorders is stressed by authors who blame impulsivity for EDs treatment failure or relapses [38].

- Strength and limits—This study relies on a large and inclusive sample size, recruited through a systematic search of well planned and carried out studies without any restriction (language, time, country). A possible limitation is that the Yale Food Addiction Scale is a self-reported tool, but, at the moment, it is the only acknowledged instrument to measure food addiction.
- Other psychiatric comorbidities were not specifically reported in all studies as well as gender composition among participants. Furthermore, patients with eating disorders were recruited in Eating Disorders Psychiatric Departments, but their medications or psychotherapy were often omitted.

## What is already known on this subject?

The concept of “Food addiction” gained more and more interest in recent years. Its neurobiology and possible comorbidity or inclusion within eating disorders are still poor examined.

## What does this study add?

This is the first systematic research, to our knowledge, that demonstrates the existence of food addiction as a separate entity from any eating disorder or other psychiatric illness. This evidence stresses the need of further studies for a better comprehension and treatment implications.

## Conclusion

Food addiction is a reality that gained more and more attention in the last years. Our work provides important evidences that FA is a separate entity from different eating disorders and afflicts each of them to a different degree. Some authors hypothesize that food addiction could have a prognostic value [26] and results from this systematic review allow the possibility to credit food addiction as worsening a comorbid eating disorder As a consequence, we strongly encourage the evaluation of the presence of food addiction in patients with eating disorders as well as in those with obesity to tailor their treatment with greater precision and address all the components that may influence patient’s response to treatment and his/her outcomes.
